# ‘*Hunger would kill us instead of COVID‐19*’: Elders' response to the pandemic in Debre Markos Town, Ethiopia

**DOI:** 10.1111/hsc.13774

**Published:** 2022-03-02

**Authors:** Anduamlak M. Takele, Messay G. Kotecho, Margaret E. Adamek

**Affiliations:** ^1^ Department of Sociology Debre Markos University Debre Markos Ethiopia; ^2^ Department of Social Work and Community Development University of Johannesburg Johannesburg South Africa; ^3^ School of Social Work Addis Ababa University Addis Ababa Ethiopia; ^4^ School of Social Work Indiana University Indianapolis IN USA; ^5^ Addis Ababa University Addis Ababa Ethiopia; ^6^ University of Gondar Gondar Ethiopia

**Keywords:** COVID‐19 pandemic, elder care in Ethiopia, Global South, hunger, institutional welfare, social isolation, vulnerable elders

## Abstract

The COVID‐19 pandemic has posed unpredictable challenges globally. Older adults are among the population groups most vulnerable to COVID‐19. Poor urban elders in Ethiopia struggle to meet their basic needs due to waning traditional familial norms of providing care for elders as a result of urbanisation, poverty and migration. The aim of this study was to give voice to vulnerable elders during the COVID‐19 lockdown in Ethiopia and to reveal their sources of support. Using a qualitative case study design, data were collected from 27 elders aged 60 and above in Debre Markos Town, Ethiopia via in‐depth interviews conducted from March–May 2020. To determine the nature of support provided for destitute elders, data were also gathered from two local officials. Narrative data were analysed using thematic analysis. Four prominent themes were identified: ‘Hunger would kill us instead of COVID‐19,’ ‘Feeling hopeless and begging to die,’ ‘We prefer social support rather than food donations’ and ‘Gratitude.’ Elders considered the practice of social distancing to fight COVID‐19 as an unwelcome luxury for people whose livelihood depends on begging and petty trade. The physical distancing programme put elders in isolation, diminishing their capacity to maintain their livelihood. Due to the increasing disrespectful attitudes towards aged people, elders felt even less valued than usual. Interactions were described as undermining, embarrassing and abusive. A special emergency fund and organised social supports are needed to minimise the effect of the pandemic on vulnerable groups like destitute elders in Ethiopia. Moreover, an institutional welfare response is needed to ensure elders can live a dignified life.

**What is already known about this topic**

- Globally, older adults are the most vulnerable group to COVID‐19 infection.
- In Global South nations, the challenge of meeting elders’ needs during the pandemic is aggravated due to the decline of traditional family care and the lack of social protection.
- Research on care and support for elders in response to COVID‐19 in Ethiopia is lacking.

**What this paper adds**

- For vulnerable Ethiopian elders, hunger was a more immediate risk than the COVID‐19 virus.
- As a result of pandemic lockdown measures, destitute elders who relied on begging and petty trade were cut off from their source of livelihood.
- The COVID‐19 pandemic has exposed the weak infrastructure of care for older adults in Ethiopia.

## 1 INTRODUCTION

COVID‐19 has brought unexpected challenges to human beings globally and its effects varied based on the socio‐economic status of particular populations. Elders are particularly affected by COVID‐19 due to preexisting chronic medical health conditions like diabetes and heart disease (Daoust, 2020; WHO, 2020). Elders need special attention during the COVID‐19 crisis, and their voices, opinions and concerns must be heard (UNFPA Global Ageing Network, 2020). Older adults living in long‐term care facilities, who live alone, or who are highly care‐dependent are especially vulnerable to COVID‐19 infection and may face barriers to obtaining accurate information, food, medication, and other essential supplies during quarantine or social distancing conditions (WHO, 2020).

According to the UNFPA Global Ageing Network (2020), although the proportion of elders in developing countries is lower than in Global North countries, the existing system for geriatric care is elder unfriendly. In general, Global South nations have the fewest health resources, limited experience caring for older patients (including few geriatric specialists), less institutional care for elders, and few public or NGO support structures for outreach, screening and community‐based care of elders. The challenge of meeting elders’ needs is aggravated due to the decline of traditional familial care systems for elders in Global South countries as a result of urbanisation, poverty, migration and HIV/AIDS (Zelalem et al., 2021). To a large extent, elders in Africa were struggling to get the necessary care and support before the pandemic.

In Ethiopia, care for elders has traditionally been provided by family, neighbours and church institutions. Increasingly, many elders are left without a caregiver and struggle to survive by engaging themselves in begging, small business activities, and other informal livelihood means (HelpAge International, 2013). In the absence of any public income support, many elders in Ethiopia are facing the challenges of homelessness, financial abuse and neglect (Kotecho et al., 2022). Ethiopia, a country with more than 5 million elders, has not yet implemented a national ageing policy or any specific entitlements for older adults.

Researchers have begun to document issues in relation to older adults and responses to COVID‐19. Using data from 27 countries, Daoust (2020) studied older people's responses to the pandemic and found that despite the greater mortality rate among older adults from COVID‐19, they were not the most compliant age group to preventive measures. Despite the geographic reach of Daoust's analysis, none of the countries studied were on the African continent. In an even broader study of the impact of the COVID pandemic on elders with data from 62 nations including three African nations, Kim and Jung (2021) reported that social distancing measures increased the mental distress of elders. While an increasing number of studies have documented the unmet needs of elders in Ethiopia before the pandemic (e.g. Abate et al., 2020; Abdi, 2012; Ayana, 2012; Chane & Adamek, 2015; Hamren et al., 2015; Moges et al., 2014; Zelalem et al., 2021), research on care and support for elders in response to COVID‐19 in Ethiopia is lacking. With the goal of shedding light on ways to protect elders from COVID‐19 infection and to ensure their basic needs are met, we examined Ethiopian elders’ responses and needs in relation to the COVID‐19 pandemic. Thus, the aim of this study was to give voice to vulnerable elders during the COVID‐19 lockdown in Ethiopia and to reveal their sources of support.

## 2 METHODS

### 2.1 Research design

Social constructivism was employed as a research paradigm due to its fit with the researchers’ stance regarding the relativity of knowledge. The social constructivist paradigm discloses the existence of manifold realities and subjective meanings (Creswell, 2014). A multiple case study design was used as this approach is best suited to depict and understand research participants’ views about their felt concerns. A descriptive case study approach is used to develop an in‐depth understanding of participants’ experiences in their context (Yin, 2012), uncovering the meaning they give to their experience of a social phenomenon (Creswell, 2014). Given the focus of this inquiry on older adults’ perspectives about their changing social context, in particular, the social distancing measures put in place in early 2020 in Ethiopia, a case study was a good fit for this study.

### 2.2 Study site

The study was conducted in Debre Markos Town, Amhara Regional State, Ethiopia. Debre Markos Town is found 300 km from Addis Ababa, the capital city of Ethiopia. Nearly half of the residents of Debre Markos Town have monthly incomes below the federal poverty rate in Ethiopia (Biyena & Beyene, 2019). Among the 7 kebeles or subdistricts (smallest government unit in Ethiopia) of Debre Markos Town, kebele 04 was selected using a purposive sampling technique. Purposive sampling is used in studies where participants are selected based on specific cases of interest (Bernard & Bernard, 2013; Rubin & Babbie, 2014).

The first author was a member of the Debre Markos University's Psycho‐social Care and Support Sub‐Committee under the university's COVID‐19 Prevention and Response Task Force. During that time, the researcher was working as a social worker/resource mobiliser to prevent food insecurity among vulnerable children, women and elders, and as a COVID‐19 Prevention Educator in the study area.

### 2.3 Sample

The target population for this study was 35 elders who had been screened for nutritional support and COVID‐19 prevention supplies such as sanitiser and masks. From this group, 27 participants were selected using the following criteria: (a) willing to take part in the study, (b) living in Debre Markos City, (c) having an active connection with the kebele 04 Women Affairs Office of the city, (d) not having permanent income/ a history of multiple sources of income, and (e) having health problems such as HIV/AIDS or a disability. The head of the Debre Markos City kebele 04 Women Affairs Office assisted with identifying potential study participants. Two experts from the East Gojjam Zone Women Affairs and the Labor and Social Affairs Office were also interviewed to provide context for the study.

### 2.4 Data collection

Data were collected after obtaining an ethics approval letter from the Office of Research, Addis Ababa University and after securing the permission of the kebele's head. Data were collected using in‐depth interviews and a document review. The study purpose and procedures were described to research participants as part of the oral consent procedures. The researchers met with the participants face‐to‐face to conduct individual interviews. Interviews were held in the kebele's office, elders’ rented homes or in Saint Michael church. Interviews were held from March–May 2020 and took 30–45 min. Interview guides were prepared in Amharic, the mother tongue of the participants. Both the interviews and the transcriptions were initiated in Amharic and later translated into English.

Several open‐ended questions and probes were used to ask study participants about their experiences with maintaining their livelihood since the start of the 2020 pandemic including the impact of social distancing measures such as mask wearing, maintaining physical distance, using hand sanitiser and lock‐down orders. Respondents were asked about their sources of support since their usual means of livelihood were severely eroded due to such measures. Finally, respondents were asked to share their perspective on changes needed to support vulnerable elders.

### 2.5 Data analysis

Data analysis procedures followed Braun and Clarke’s (2006) six steps of thematic data analysis: (a) familiarising oneself with the data, (b) generating initial codes, (c) looking for themes, (d) reviewing themes, (e) defining and naming themes and (f) producing the report. The issue of credibility in qualitative research concerns the extent to which the data gathered reflects the perspectives and circumstances of the study participants. To enhance the trustworthiness of qualitative data, Anney (2014) recommends approaches such as prolonged engagement in the field, peer debriefing, triangulation, member checking and providing ‘thick’ descriptions. To establish the credibility of the data, triangulation was used in this study by collecting data from three sources: the older adults themselves, local government officials, and a review of pertinent policy documents. In addition, as the themes were identified from the narrative data, peer debriefing was used to ensure agreement on the identified themes. Researchers contacted the study participants for member‐checking to give them the opportunity to review a first draft of the study's findings.

### 2.6 Ethical considerations

To ensure the ethical nature of the study, data collection was carried out using voluntary participation, informed consent, and privacy protection with the research participants. The participants were all adults, aged 60 and above. They were each given information about the nature of the research, the potential benefits, risks, and ways to withdraw from the study. Oral consent was obtained from all participants.

## 3 FINDINGS

Twenty participants were female and 7 were male. They ranged in age from 60 to 82 with an average age of 72. About half (*n* = 13) were homeless and lived outside in church courtyards. All were followers of Orthodox Christianity. Most of the participants were divorced. In terms of health status, all of the participants were suffering from illnesses like HIV/AIDS and various forms of physical disability which exacerbated their vulnerability to COVID‐19.

As Table 1 shows, most elders in the study area earned their living from begging and petty trade. The pandemic has been a particular challenge for them since their business activities were interrupted to avoid COVID‐19 infection. Wheat flour and teff (grain from an annual grass native to Ethiopia) were distributed to elders according to family size. Accordingly, a household with three or more family members received 21 kilos of teff and 10 kilos of wheat per month.

**TABLE 1:** Social‐demographic characteristics of participants (*n* = 27)

No.	Pseudo names	Sex	Age	Kebele	Health condition	Living arrangement	Marital status	Livelihood
1	Almaz	Female	65	01	Visual impairment	Living in a rented Keble house	Widow	Petty trade
2	Atnaf	Female	60	01	PLWHA	Rent house	Single	Petty trade
3	Alex	Male	75	04	Visually impaired	Rented house	Unknown	Begging
4	Alemesh	Female	78	04	Physically disabled	Homeless	Unknown	Selling vegetables
5	Addisalem	Female	71	03	Physical disability	Homeless	Unknown	Begging
6	Banchi	Female	78	05	Visually Impaired	Homeless	Unknown	Begging
7	Belaynesh	Female	79	04	PLWHA	Homeless	Unknown	Begging
8	Bezawit	Female	73	05	PLWHA	Homeless	Unknown	Begging
9	Belaynew	Male	75	04	Visually impaired	Homeless	Divorcee	Begging
10	Beletech	Female	65	01	PLWHA	Keblele house	Divorcee	Begging
11	Dagne	Male	80	04	PLWHA	Homeless	Unknown	Begging
12	Ebsete	Female	80	04	PLWHA	Homeless	Unknown	Begging
13	Kebebush	Female	82	04	Leg infection	Homeless	Unknown	Begging
14	Kidist	Female	67	05	Diabetics	Rented house	Single	Laundry lady
15	Marta	Female	60	05	PLWHA	Keble house	Divorcee	Selling Kolo/Snacks
16	Shetaye	Female	70	05	Not known	Keblele house	Unknown	Begging
17	Tigist	Female	67	04	PLWHA	Keble house	Widow	Petty trade
18	Tirunesh	Female	74	04	Physically disabled	Homeless	Divorcee	Begging
19	Tebelete	Female	72	03	PLWHA	Rented house	Unknown	Selling Injera
20	Tringo	Female	75	04	PLWHA	Homeless	Unknown	Begging
21	Wondemenew	Male	74	03	Visually Impaired	Homeless	Unknown	Begging
22	Wassihun	Male	67	05	PLWHA	Homeless	Unknown	Begging
23	Wondemu	Male	70	04	Visual impairment	Living with his grand child	Divorcee	Begging
24	Yalemwork	Female	68	04	Physically disabled	Rented house	Unknown	Lottery seller
25	Yigardush	Female	69	03	PLWHA	Rented house	Unknown	Selling bread
26	Yeshiwas	Male	73	05	Physical disability	Rented house	Unknown	Begging
27	Zemenay	Female	77	05	PLWHA	Homeless	Unknown	Begging

Abbreviation: PLWHA, people living with HIV/AIDS.

### 3.1 Themes

The narrative data from the in‐depth interviews with the 27 elders revealed four prominent themes relating to hunger, hopelessness, lack of social support and gratitude.

### 3.2 ‘Hunger would kill us instead of COVID‐19 pandemic’

According to the study participants, the COVID‐19 pandemic was not the real challenge by itself for elders. For those with no permanent source of income and no supportive social network, their biggest challenge was getting adequate food to survive and for those who were housed, money to pay for rent. Their source of livelihood was highly endangered by the country's lockdown and stay‐at‐home measures during the pandemic state of emergency.

Atnaf, age 60, expressed concern with maintaining her livelihood:

*For me, whether you believe it or not, I am not afraid of the new pandemic that people call COVID‐19. My day and night nightmare is the issue of getting food and covering my cost of medical care services. Before the outbreak of COVID‐19, I was able to feed myself and my three children from the money I get from my job –selling vegetables in the market. Now, because of business shutdown associated with the pandemic, I am just simply sitting and waiting for God’s miracle to collect my daily food. I don’t know how other people reacted towards the pandemic but for me, hunger will quickly kill us before COVID‐19 if someone will not take care of our daily meals and other basic necessities that are deemed necessary for someone’s survival. Health care services for patients like me are temporarily interrupted since every medical personnel are busy in the fight against COVID‐19. In this situation, if something happens, we all are dying without getting medical attention. What an unfortunate luck we have?!*

Banchi, age 78, shared:

*I am visually impaired and earning my living by engaging myself in begging activities. This boy is my grandson, and he is the one who is responsible for taking care me. He is not lucky because he is taking care of me instead of going to school. I am living in my son’s rented home. My son is a daily labourer, and he is losing his job associated with the outbreak of the pandemic. Currently, we are short of food and other lifesaving supplies let alone buying masks and other sanitisers to prevent COVID‐19 infection. That is a secondary issue for us. What bothers us is ways of getting food items to eat, nothing else*.

*Let me ask you one question, child?! How dare you teach me about COVID‐19 prevention measures such as buying and wearing mask while I am in need of food to fight hunger? That is irony, boy! You see, that is the difference between the well‐off and the poor. I am not worried to be infected by COVID‐19 virus, but I am worried to be called a man who died of hunger in a place where plenty of resources are available. As you know, Gojjam is considered as the breadbasket of Ethiopia; it is feeding the rest of Ethiopia but here people like me are struggling to defeat hunger. It is highly paradoxical*.

Almaz, 65, also considered the government stay‐at‐home measure as a luxury that lacked insight about the preexisting inequality and its repercussions on people whose survival largely depends on the informal sector. According to Almaz, lock‐down measures should take place *after* food and other necessary items are distributed to the poor in a coordinated manner until the lockdown measure lifts. She highly criticised the current stigma‐oriented mode of providing support.

The physical distancing programme introduced to contain the pandemic diminished elders’ capacity to get the support of others to perform their daily activities. Tigist, 67, shared her resentment towards physical distancing and its unintended effect on the social interaction of vulnerable elders:

*Back in days, we elders have got the chance to get any form of assistance from anyone but after the introduction of physical distancing and self‐isolation measures against COVID‐19, people are afraid to lend a hand even if we seek their assistance. Such problem is even worse when you are suffering from sight problem. The norm of assisting visually impaired people is deteriorating due to fear of COVID‐19 infection. People are afraid to help persons with disabilities to avoid contamination. Elders like me need emotional as well as material support in a pandemic like this. I think it is very challenging to lead one’s life if someone who is able‐bodied is not giving support for them*.

### 3.3 ‘Feeling hopeless and begging to die’

Due to the deteriorating capacity of families to care for elders and the emergence of disrespectful attitudes towards aged people in the town, elders are not seen as valuable. Interactions were described as undermining, embarrassing, and abusive. Elders depicted their future as full of darkness as their relatives were not able to provide the type of care they needed. Wondemnew, aged 74, shared his concern regarding the prospect of having a caring significant other:

*In my productive age, I had the chance to get any assistance from my significant others but now I think I will no longer be lucky to do so. I think I am useless for my town and my relatives. I am not blaming people for not lending me a hand, but I am complaining about my destiny. Life in the town makes people rude and most of the time people are bored to listen to elders’ concern. Even they do not dare to give you their ear. What a cursed time?! My relatives have no interest to pay a visit to me. I have no one to rely on. I don’t think the future is bright for us. It would be gloomy. I am just begging God to invite me to live in his kingdom, nothing else*.

Similarly, Yigardush, aged 69, agreed about what it means to be a poor elder in the eyes of city residents:

*For me, what is called elderly and the associated grace with it in the past is almost vanishing in the city now. As you might observe, elders like me are not respected and are considered as a burden in society. My friends have told me that youth are doing their best to show their respect for us. But I doubt that. What makes life miserable is living a life that cannot be valued by others and losing your place in society where you belong. I think I prefer to die like this than accepting many more disrespectful signs ahead. I am tired of living like this. I wish I could die today. I do not see any hope in the future. I am hopeless*.

Yeshiwas, age 73, shared a different perspective reflecting his view of ageing as part of the challenge of life:

*I don’t see ageing as a sign of disrespect rather it is a taste of humanity. I am thinking that because I have seen so many life vicissitudes. It is when you get older, you get the opportunity to question yourself; what is my calling? What do I do to fulfil my mission in this world? I think, we, elders need to defeat our feeling of hopelessness by preparing ourselves to do something good for each other and the rest of the world. We need to show the youth the right way of respecting us. This could happen by sharing our wisdom with them. The youth has no chance to learn the gift of ageing. We need to establish such knowledge transfer. In that way, we find the meaning of life and purpose of living the life we cherish. Personally, I don’t blame myself for being an elder person rather I am feeling guilty of not using my capacity to teach the youth about ways of learning wisdom from elders like me and not answering the question of ‘what blessings are there in respecting elders of the community?’ to the youth. I think we need a perspective like that*.

### 3.4 We prefer social support rather than food donations

Some elders were concerned about the impact of pandemic‐related isolation on their mental health. Belaynesh, 79, expressed her resentment as follows:

*For me, it would be better to embrace death rather than living a disrespectful life like this. I was a respected woman during my prestigious time. I had my husband, I had wealth, and I had children in my home. Now, I am lonely and living in the compound of St. Michael Church. You know what is killing me inside? You, you have a respected job, your family and beautiful home, more importantly, you have friends in your circle. But I have no one on my side. It is my inability to get such love and affection that starts killing me inside. We prefer greetings rather than eating*.

Yalemwork, age 68, explained that COVID‐19 disrupts various social interaction means like idir, wedding, holidays, and funerals that could be a source of social interaction for elders:

*Before COVID‐19, even though we have no place in the prestigious section of any festival like wedding, we were able to attend ceremonies and had the opportunity to talk with community members. But now, thanks to COVID‐19, such means of social gathering are prohibited. Hence, we lost both means of collecting food and preventing social isolation. It is very scary to live a life that is estranged‐‐the one you have not socialised to live*.

Zemenay, age 77, likewise pointed out how much she missed her old way of life:

*For me, it is too scary not to have physical proximity, not to talk with community members, have a visitor. I think COVID‐19 prevention instructions like physical distancing, prohibition of social gathering like wedding is odd for us. We were not socialised to live a life which is full of misery. You have no idea how much I missed the old way of life‐‐attending funerals, wedding ceremonies, having greetings/handshaking/chuckle, kissing and the like. I think people around the city are not quite familiar with the new normal. We used to live a life which is full of handshaking and face‐to‐face interaction. I don’t think we could make it unless God has to do something to eradicate such pandemic*.

Bezawit, aged 73, envisioned the way forward to address the multifaceted needs of elders:

*The problem of vulnerable elders like me needs a structural solution not occasional subsistence support from philanthropists. It demands institutional and policy response. I think emergency fund for elderly care during disasters like COVID‐19 needs to be in place. And other income‐generating mechanisms like elder’s business entrepreneurship packages need to be solicited if concerned bodies are to ensure our dignified life and death. Even, the culture of Aqolequay‐‐feeding the elderly in times of difficulty‐‐has to start in the town. I know such forms of solidarity are widely practiced in rural parts of Gojjam society. Such social support mechanisms should be begun in the town or else we will continue living a miserable way of life*.

Aqolequay refers to an Ethiopian Ubuntu which is a norm of reciprocity in times of difficulty. In the culture of Gojjam society, individuals are expected to share in the sorrow and happiness of fellow citizens by contributing to the cost of wedding ceremonies and funerals and by assisting with harvesting crops and supporting the needy including vulnerable elders.

According to an expert from the Labor and Social Affairs Office, the issue of elders’ vulnerability needs a policy and institutional response. Caring for vulnerable elders should not be a seasonal campaign of humanitarian organisations and individual philanthropists. He suggests that the welfare regime needs to be changed from residual to institutional or developmental to honour elders’ contributions and allow them to live a dignified life. Such concern was echoed by the Head of the Women Affairs Office:

*As you can see, occasional support for vulnerable elders in our town is done haphazardly and in an informal way. For me, such welfare approach is dehumanising and not transforming the lives of our senior citizens. We need to change the way we care for elders. We have been using such residual approach for years without witnessing a positive change in the lives of our poor elders. It perpetuates dependency and deteriorates the value of our elders*.

Existing evidence confirms the absence of an independent policy that specifically addresses elder care and support in Ethiopia.

### 3.5 Gratitude for occasional support as a life‐saving scheme

Despite the challenges they face, most participants were grateful for the support they were provided. Accordingly, Ebsete, aged 80, expressed her gratitude:

*I think you reach out to us in the right time. We are facing starvation due to our inability to feed ourselves. The kind of support that we are receiving is a life rescue support. I am calling it like this since it rescued me from hunger for the time being. As you can see, everybody is not willing to support us even if he/she is kind to do so. The time is very scary! People are no longer supporting us because they themselves may be running out of resources. The pandemic has become a huge burden for people like us. We don’t know what will happen in the future. For the time being, we are happy to get your support. I think your 21 Kilo Teff and 10 Kilo Wheat flour will feed us for a month and as I heard from my friends such form of assistance will continue till the end of lockdown measure. That would be nice for us to sustain our lives*.

Similarly, Tringo, aged 75, also expressed gratitude:

*You have no idea how much it means for the forgotten people like me. It means a lot for us. It really gives hope to live our lives. I would call it your generosity as something life‐ sustaining. What would we do without your support? We would all die, no option at all. Thank you so much and may God bless you. We owe you and we will never forget what you have done for us*.

A few other elders also expressed appreciation for the measures taken by concerned bodies to support vulnerable elders during the COVID‐19 lockdown. According to Tibelete, age 72:

*I* *was running out of food and I was not able to buy any food item, but you reached out to poor elders like me. Thank you so much! May God bless you*

For Tigist, age 67, the food donation was critical:

*For me, the food donation is everything. I was worried about the means of getting food in a scary situation like this. How can any hungry person beg to get his daily meal while people are locking their door to avoid COVID‐19 infection? I do not know. But here I get it through your kindness. I am thankful for that*.

For Tirunesh, age 74, the food donation enabled some poor elders to stay in their rented house and prevent them from abuses inflicted on homeless persons:

*I think the food donation will be an asset for other elders who are struggling to cover the cost of both food and rent. At least, elders who planned to spend their little money to buy food will use their money to cover their cost of rent. In this case, they are rescued from potential forms of abuse as a result of being homeless. As you can see, I am a homeless elder and I know what it means to be homeless. Being homeless means powerlessness, vulnerability to sexual abuse, verbal abuse, and the like. I think it is a good opportunity for other elders to stay at their rented house. Hence, the food donation is lifesaving*.

## 4 DISCUSSION

For the Ethiopian elders in this study, hunger was a more immediate risk than the COVID‐19 virus. In the absence of institutional supports, the response to the needs of vulnerable elders in Ethiopia during the pandemic has been insufficient. With the shutdown of businesses during the lockdown period alongside the breakdown of the norm of reciprocity, vulnerable elders were left with no means to support themselves. As a survival strategy, elders turned to prayer and waiting on God's miracle to meet their daily needs. According to a UNPFA (2020) policy briefing, many elders need nutritional support and other types of practical assistance to cope with the challenges of the COVID‐19 pandemic. As in other parts of the world, ‘COVID‐19 countermeasures put older adults at higher risks of abuse and neglect through interactions with factors at multiple eco‐systemic levels: individual (older adults), caregiver, care‐giving, and structural’ (Yunus et al., 2021, p. 1).

In addition to hunger, elders faced social isolation due to the social distancing restrictions and lockdown measures. Study participants expressed a preference for greetings, or social support, over even material support to enhance their well‐being. Likewise, Teka and Adamek (2014) found that social interaction was critical for Ethiopian elders’ emotional well‐being. Elders in this study pointed out that an indigenous mutual support system like *Aqolequay* can be a safety valve to help them endure the pandemic season. While still operational in rural areas, such mutual aid support systems are less likely to be operating in urban areas.

In the absence of a national policy that entitles elders to social protection and various basic supports, the issue of elder care and support is left to individuals and families. Specific COVID‐19 emergency funds for elder care and other urban safety net approaches for vulnerable elders need to be prioritised to improve the lives of poor elders. The study confirmed elders’ vulnerability to homelessness and their exposure to various forms of abuse. Hence, vulnerable elders need living options such as a community‐based or institutional care centre to meet their basic needs and to prevent abuse. As noted by HelpAge International (2013), many urban elders in Ethiopia support themselves by engaging in the informal sector through begging or petty trade. Elders were unable to afford masks or sanitisers to prevent COVID‐19 infection. Moreover, elders were unaware of the means of COVID‐19 transmission as the pandemic was not their primary worry.

While the elders in this study needed emergency food donations, they would have preferred an approach that empowers them rather than one that depicts them as welfare recipients and a burden on society. Institutional welfare systems provide support for all those who are eligible irrespective of race, gender, disability and other identity indicators. The researchers strongly support a welfare scheme that respects the dignity and worth of each human being. Hence, a long‐term institutional response from the central government is needed in the study area and throughout the nation. A community that values the contribution of its older citizens is highly needed and thus an entitlement system should be in place to ensure elders’ well‐being. In addition to food distribution, a special emergency fund should be introduced to minimise the effect of the pandemic on vulnerable groups like destitute elders in Ethiopia.

### 4.1 Study strengths and limitations

This qualitative inquiry was able to give voice to vulnerable elders living in Debre Markos Town, Ethiopia, revealing their struggles with food insecurity as a bigger concern than the COVID‐19 pandemic. Although grateful for handouts of food and other necessities, the study participants shared their perspectives about the inadequacy of supports for older adults. Essentially, the COVID‐19 pandemic exposed the prevalence of an ageist culture in Ethiopia that blocks the development of meaningful social protection for older adults, leaving them in extreme financial strain‐‐pandemic or no pandemic.

Methodologically, the study was limited to a non‐representative sample in one city which thereby excludes the voice of elders towards COVID‐19 in other parts of Ethiopia. Most Ethiopian elders live in rural areas (Central Statistical Agency, 2012), and thus research is needed to shed light on the impact of the pandemic on the well‐being of rural elders. A nationwide study using a mixed method approach is needed to more accurately depict the magnitude and nature of elders’ vulnerability to COVID‐19 in Ethiopia. The data for this study were collected during the early months of the pandemic in Ethiopia when social distancing and stay‐at‐home orders were at their peak and thus elders’ perspectives later on the pandemic when such orders were relaxed are not portrayed.

The COVID‐19 pandemic has exposed the weak infrastructure of care for older adults in Ethiopia. Even in resource‐rich countries like the U.S., the pandemic revealed increased vulnerability to negative outcomes for older adults due to the intersecting dynamics of ageism, racism, and classism (Morrow‐Howell et al., 2020). Cox (2020) explains that although old age is a significant contributor to vulnerability to the negative outcomes of COVID‐19, it is the interaction of age with other factors such as chronic health conditions and poverty that makes age such a strong determinant. Likewise, Reynolds (2020) describes the pandemic as a ‘focusing event’ that uncovered ‘the extreme social consequence of ageism’ for the older population (p. 499). In Ethiopia, the lack of institutional supports for vulnerable elders meant not only social isolation and the associated mental stress during the pandemic, but the likelihood of not having enough food to eat.

Various philanthropists in Ethiopia have been providing care and support from a humanistic perspective and not from the view of older adult care as an obligation of the state. Although elders were grateful for the occasional support during the COVID‐19 lockdown, this residual approach was viewed by elders as dehumanising. An empowering model that gives elders the chance to live a dignified life is needed rather than the current approach that represents elders as unproductive and as a societal burden. Unlike Global North countries, many Global South nations lack a policy framework that obliges states to provide various care and support mechanisms for older adults. This can be a useful lesson for Ethiopia, a country whose policy framework largely ignores the issue of elders.

Education and training for policy makers and government officials on alternative models of support for elders is crucial and timely. The haphazard nature of food distribution to elders during the pandemic highlighted the weaknesses of a residual policy. Hence, policy advocacy is needed to support a functional ageing policy in Ethiopia that views support for elders as a state obligation. As Carrieri et al. (2020) argue, the public sphere must be shaped ‘in ways that allow [us] to recognise both the vulnerabilities and potentialities of all generations. One way to do so is to respect the individual person, going beyond categories such as age or age groups, and to promote intergenerational equity and solidarity by fostering connectedness across different generations’ (p. 3).

## 5 CONCLUSION

Professionals in social work, public health, nursing and other helping professions need to be mobilised to provide care and support for vulnerable elders. Though writing about the U.S., Cox’ (2020) statement rings true worldwide, ‘Social work interventions to reduce the systemic disparities impacting older adults in the midst of the Covid‐19 pandemic are essential’ (p. 620). In Global North countries helping professionals with geriatric training are available to provide psychosocial care and support to elders at risk of physical or mental distress. As social work educators, we recommend the establishment of a geriatric health service system including mental health services for vulnerable segments of the Ethiopian population including elders. We strongly advocate for the establishment of community‐based elder care centres and various income‐generating mechanisms to help elders remain self‐reliant and live dignified lives.

## CONFLICT OF INTEREST

The authors declare that there is no conflict of interest to disclose.

## AUTHOR CONTRIBUTION

AT and MK conceptualised the study design. AT and MK contributed to data collection and analyses as well as the original drafting of the manuscript. MK provided supervision of data collection and analyses. MA contributed to the literature review, presentation of findings and final drafts of the paper. All authors critically revised the manuscript and accepted its final form.

## Data Availability

The complete set of narrative data for this study is being stored by the first author, AT.
